# Integrating digital and field surveillance as complementary efforts to manage epidemic diseases of livestock: African swine fever as a case study

**DOI:** 10.1371/journal.pone.0252972

**Published:** 2021-12-31

**Authors:** Michele Tizzani, Violeta Muñoz-Gómez, Marco De Nardi, Daniela Paolotti, Olga Muñoz, Piera Ceschi, Arvo Viltrop, Ilaria Capua

**Affiliations:** 1 Institute for Scientific Interchange Foundation, Torino, Italy; 2 SAFOSO, Liebefeld, Switzerland; 3 Section of Epidemiology, Vetsuisse Faculty, University of Zurich, Zurich, Switzerland; 4 One Health Centre of Excellence, Gainesville, Florida, Unites States of America; 5 Department of Environmental and Global Health, College of Public Health and Health Professionals, Gainesville, Florida, United States of America; 6 Estonian University of Life Sciences, Tartu, Estonia; University of Lincoln, UNITED KINGDOM

## Abstract

SARS-CoV-2 has clearly shown that efficient management of infectious diseases requires a top-down approach which must be complemented with a bottom-up response to be effective. Here we investigate a novel approach to surveillance for transboundary animal diseases using African Swine (ASF) fever as a model. We collected data both at a population level and at the local level on information-seeking behavior respectively through digital data and targeted questionnaire-based surveys to relevant stakeholders such as pig farmers and veterinary authorities. Our study shows how information-seeking behavior and resulting public attention during an epidemic, can be identified through novel data streams from digital platforms such as Wikipedia. Leveraging attention in a critical moment can be key to providing the correct information at the right moment, especially to an interested cohort of people. We also bring evidence on how field surveys aimed at local workers and veterinary authorities remain a crucial tool to assess more in-depth preparedness and awareness among front-line actors. We conclude that these two tools should be used in combination to maximize the outcome of surveillance and prevention activities for selected transboundary animal diseases such as ASF.

## Introduction

African Swine Fever (ASF) is a transboundary animal disease that affects wild and domestic pigs, and it is characterized by a high case fatality rate [1]. ASF is present in several European, African, and Asian countries, its impact on global markets can potentially be catastrophic, threatening the economy from local to global level [1], jeopardizing pig industries [2], and negatively affecting socio-economic factors [3]. The World Organization for Animal Health (OIE) and the Food Agriculture Organization (FAO) of the United Nations recognize ASF as a global threat that should not be underestimated and that requires global long-term commitment [4]. Awareness campaigns are considered essential for the prevention and prompt intervention of the veterinary health authorities [5]. The unavailability of a vaccine or treatment makes awareness programs and mass culling among the few options available for disease prevention and control [6].

Despite decades of international control efforts, the disease is still spreading in various regions of the world with different epidemiological dynamics [4]. These are strongly influenced by the natural environment that impacts the density of susceptible species and vectors (i.e. ticks) but are also significantly affected by human behavior [7]. In particular, human behavior plays a key role in the transmission and geographic spread of the ASF virus [8, 9] through infringement or low compliance with biosecurity and preventive measures, movement of contaminated fomites, and/or products, and underreporting of ASF suspected cases [6, 9].

The global spread of ASF together with the heterogeneous epidemiological dynamics, lack of treatment and vaccine, inappropriate human behavior, and biosecurity measures make the control of ASF a complex scenario [4] that calls for novel solutions to preparedness and surveillance that could go beyond non-traditional methodological approaches.

ASF was first reported in Estonia in wild boars in 2014 [10] and domestic pigs in 2015 [11]. The incursion of the ASF virus to domestic pigs was very likely due to the introduction of contaminated fomites by people (e.g., clothing, vehicles, feed, bedding material) to pig farms as a result of insufficient biosecurity measures [11]. Estonia has been active in field and research programs on ASF prevention and control and has developed collaboration programs between the farmers and the official veterinary services. The Estonian government has invested efforts in raising awareness of ASF among the general population and animal-related target groups, such as pig farmers and hunters [12, 13]. Moreover, Estonia has been selected as a case study due to its advanced digital environment [14] and because of the recent circulation of ASF in both wild boar and domestic pig populations [11].

In particular, we propose an integrated approach that combines survey data and digital trade data. Survey-based research and digital data-based research are two different methodologies extensively used in both health and social science. The two paradigms differ in both the data collection method and the data analysis [15, 16], nonetheless, the integration of these two approaches has been proven useful in studying human behavior [17, 18]. In this work, we aim to monitor public awareness and preparedness of the veterinary health authorities and field workers based on a combination of non-traditional data sources and survey-based methodologies.

Public awareness is key to developing effective communication campaigns and preventing the introduction or mitigating the spread of the disease in any given area. This phenomenon can be monitored through the exposure of the public to the news resulting in proactive information seeking, as measured for example through Wikipedia pageviews. In this case, information-seeking behavior is intended as the deliberate process of individuals to actively gain new knowledge by searching for information on a specific topic [19]. The use of Wikipedia pageviews as a proxy for measuring the collective attention to the outbreak has been already successfully adopted in other contexts, focusing on human diseases (e.g., epilepsy, asthma) [20, 21] and some zoonotic diseases, (e.g., Zika, H1N1, and SARS-CoV-2) [22–24]. Furthermore, surveys can be adopted to assess the level of preparedness of local animal health authorities and field workers who are at the frontline of disease containment and prevention [25–27]. Our proposed methodology aims at combining the insight provided by digital data to assess information-seeking behavior and awareness in the general population (with a focus on Europe) with a field survey-based study in Estonia targeting relevant stakeholders for ASF (pig farmers and veterinary authorities). The objectives of the surveys in Estonia were to explore the use of digital sources by pig farmers, to collect information on the ASF communication strategy of veterinary authorities, and to explore the opinion of the veterinary authorities on the level of ASF awareness in Estonian farmers and hunters. We believe this integrated approach, when regularly implemented, could provide better insights on the level of attention of the public towards a specific hazard and risk allowing better communication strategy and ultimately, improving risk management strategies.

## Material and methods

In this work, we adopt a two-fold approach considering both the general public and the stakeholders’ perspectives by monitoring digital sources, such as news and Wikipedia pageviews, and implementing field surveys, respectively. For digital data we applied the methods previously validated by literature [28] for monitoring information-seeking behavior among the general population in response to news exposure, focusing on ASF. The stakeholder component was assessed through two field surveys in Estonia focusing on veterinary authorities and farmers to assess the level of preparedness towards ASF. Finally, we performed a topic modeling analysis on the news content to qualitatively assess the information exposure of both the general population and stakeholders.

### Data description

We considered two main digital sources: Wikipedia, and reports from online news outlets. The data collection focused on ASF-affected countries in Europe (Belgium, Czech Republic, Estonia, Italy, Latvia, Lithuania, Poland, Romania, Ukraine), and Asia (China, India, South Korea). We considered the period between January 2015 and May 2020, since the Wikipedia pageviews data were not available before. We extracted ASF outbreaks data from the official reports of the Animal Disease Notification System (ADNS) for European countries [29], and the OIE update reports on ASF for Asian countries (Situational Updates of ASF in Asia and the Pacific—OIE—Asia).

We used the Wikipedia application programming interface (API) [23] to collect the number of visits per day to Wikipedia articles, i.e., click rates or pageviews. This number was normalized with the total monthly access to Wikipedia from each targeted country. We selected the Wikipedia articles specific for ASF, using the corresponding Wikipedia page name, namely ‘African_swine_fever_virus’, and the respective translations for the languages of interest, see S1 Table. For most of the countries considered, the language is highly indicative of the location. On the other hand, we introduced a weighted normalization factor for the number of views to account for the multilingualism of some of the countries, like Belgium and India. The weighted normalization was performed considering the total number of accesses to the main Wikipedia page in each language, i.e., a Wikipedia project (p) (S1 Table). More specifically, we weighted the number of daily accesses to a single article in a specific language, given by *S*_*p*_*(d)*, with the total number of monthly accesses from a country *c*, to the related Wikipedia project *T*^*c*^_*p*_*(d)*. Hence, the daily pageviews from a given Wikipedia project and country are described by Eq (1):

$$y_{a,p}^{c}\left(d\right)=\frac{S_{p}\left(d\right)T_{p}^{c}\left(d\right)}{\sum_{c}^{T_{p}^{c}}\left(d\right)}$$ (1)

The denominator is the total number of views of the Wikipedia-specific project. The total volume of views at day, *d*, from a country, *c*, is then given by the sum over all the articles and projects, *p*, given by Eq (2).

$$y^{c}\left(d\right)=\underset{a,p}{\sum }y_{a,p}^{c}\left(d\right)$$ (2)

We collected news from the Global Database of Events, Language, and Tone, (GDELT) project [30]. GDELT is a high-resolution open data platform that monitors news worldwide in over 100 languages, translating and processing them to identify events, people, organizations, locations, themes, languages, and original Uniform Resource Locator (URL) to the article page. The GDELT database is available in Google BigQuery (https://cloud.google.com/bigquery), and data is collected using the query described in S1 Text. We considered all the news published online between 2015/02 and 2020/05 mentioning ASF, for a total of 107,547 articles distributed among the countries as shown in S1 Table. The GDELT database offers many descriptors for each article, in particular, we considered the following entries: the URLs of the articles, the publication date, the language in which the article was written, the locations, and the topics mentioned in the article. To characterize the timeseries for each country, we counted the number of articles per date that are in at least one of the main spoken languages in the country and mention the country either in the location entry or in the topic entry of the GDELT dataset. Finally, to implement the topic modeling analysis we adopted the summary of the full text of each article retrieved from the URLs through the newspaper python library (https://github.com/codelucas/newspaper) and translated it to English using the Yandex translate API (https://yandex.com/dev/translate/).

### Statistical analysis of digital data

To analyze the correlation between ASF media coverage in each country and online users’ collective response as measured through the Wikipedia pageview, we introduced two regression models as shown in Eq (3):

$$\begin{array}{l} modelIy_{t}=\alpha_{1}news_{t}+u_{t}\\ modelIIy_{t}=\alpha_{1}news_{t}+\alpha_{2}newsMEM_{t}+u_{t} \end{array}$$ (3)

where *y*_*t*_ is the number of country-specific Wikipedia pageviews and *u*_*t*_ is the error term, and the independent variables are either the news volume or the news volume plus a memory term. The first model is a simple linear regression, while the second one includes a memory kernel to account for “memory effects” (e.g., loss of interest) in the public response to media coverage. In the latter we weighted the cumulative news articles volume time series with an exponential decaying term [28] as shown in Eq (4):

$$newsMEM=\sum_{\Delta t=1}^{\tau }e^{\frac{-\Delta t}{\tau }}news\left(t-\Delta t\right)$$ (4)

where τ is a free parameter that sets the memory timescale. We tuned τ in the range of [1,60] optimizing the results of the linear regression for the adjusted R^2^, and showing only the best results.

The analysis was carried out using the python stats models library [31], more details on the diagnostics for the two models can be found in the S1 Fig.

Finally, to qualitatively explore the content of the digital news, we analyzed the prevalent topics in the news through an unsupervised topic modeling approach [32]. Topic modeling is a statistical method that is particularly effective for classifying, clustering, and arranging textual data in latent themes. It has been extensively applied in the literature to extract groups of coherent information from a list of documents [22, 32–34]. We used a well-known probabilistic framework, the latent Dirichlet allocation (LDA) [35]. We cleaned and lemmatized the text using the python “spacy” library [36], while the number of topics was chosen through a grid search of the parameter for the Latent Dirichlet Algorithm from the scikit-learn python library [37]. All the authors (the majority of which have a background in veterinary sciences) were involved in the qualitative annotation of the resulting topics. Topic analysis was performed on Estonian news. We focused on this country to qualitatively assess the coverage of the problem on the population level and compare the results with the more in-depth analysis carried through the field surveys described in the next section.

### Surveys in Estonia

Two questionnaires were designed, one targeting pig farmers and one targeting risk managers within the veterinary authorities. Both questionnaires were designed and piloted in English and can be found in S1 and S3 Files of the supporting material. The questionnaire for pig farmers was translated into Estonian by collaborators from the Estonian University of Life Sciences (Estonia) and aimed at collecting information on demographics, type of production, experience on ASF, and perceptions related to ASF, use of social media, and the implementation of the ASF risk management strategy, using closed-ended questions. The questionnaire for pig farmers was delivered at the end of five focus group discussions that were part of a separate study, organized and implemented by the Estonian University of Life Sciences (Estonia), where farmers were asked to complete the questionnaire independently. Managers of commercial pig farms were recruited for the focus groups following a convenient sampling.

The questionnaire for the veterinary authorities, formed by open-ended questions was delivered in English, and it was designed to collect information on the coordination practices related to the national risk management of ASF, the involvement of farmers and wild boar hunters in risk management strategies, and the national communication strategy on ASF. The questionnaire was delivered through both face-to-face and phone structured interviews. Veterinary authorities that were part of our network were invited to participate in this study and thus, they were selected following a convenient sampling.

Since the survey did not include active sampling in animals/humans and no personal information of respondents was collected, it was not necessary to request Ethics committee approval. Before starting the interview, a confidentiality statement was agreed upon with all participants and, after giving consent, the interview was conducted. The data collection process for both target groups in Estonia (pig farmers and veterinary authorities) took place from November 2019 to February 2020 and it was interrupted due to SARS-CoV-2 restriction.

Survey data were gathered in Excel©. A descriptive analysis of all the questions was conducted for both questionnaires. Questions that are included in this study are indicated in the questionnaires in the supplementary documentation.

## Results

### Digital news, Wikipedia pageviews, and public awareness

**Fig 1:**
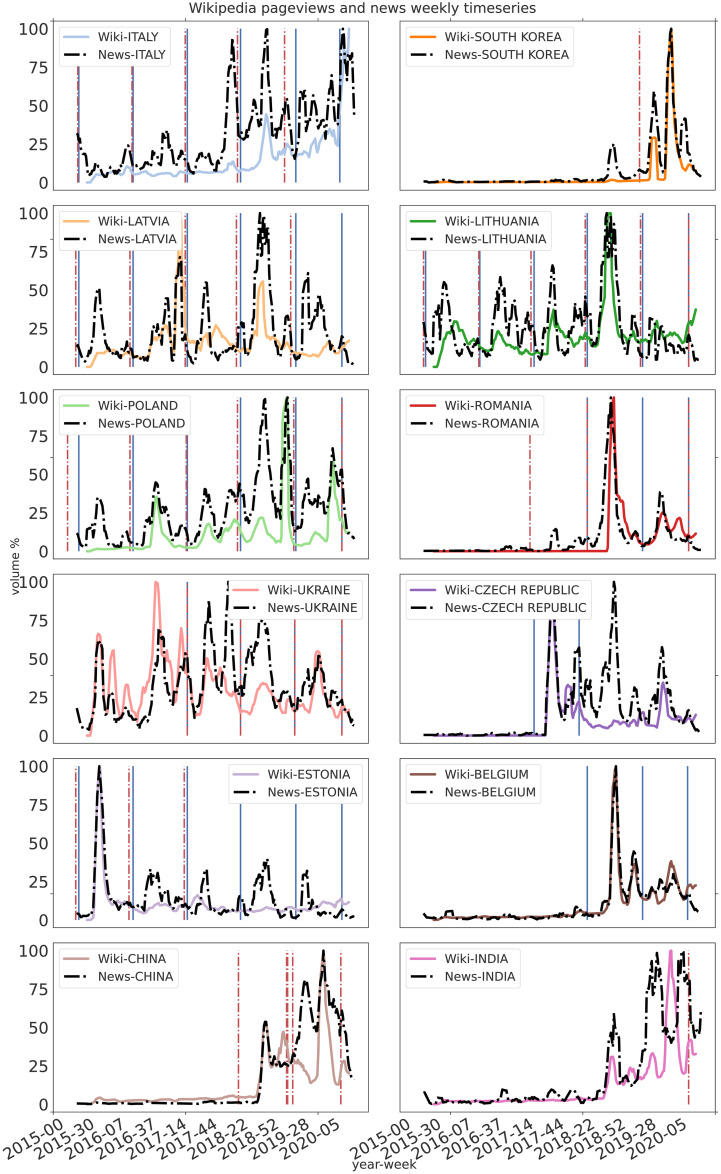
Weekly moving average of Wikipedia page view count, colored-solid lines, news volume, dotted-black lines, and ASF surveillance cases reports for domestic pigs, dashed-red vertical lines, and wild boars, solid blue vertical lines.

In Fig 1 we show the comparison between weekly aggregated normalized volumes of news and Wikipedia pageviews, for each country compared with the reports of ASF outbreaks in the selected countries for domestic pigs and wild boars. For clarity, we display the moving average of both the timeseries with a window of 6 weeks computed with the *pandas* python library [38].

The temporal profile of the two digital signals is very similar, showing synchronization of the time-series for most of the countries, as confirmed by the correlations in Table 1. Notice that the information-seeking behavior decreased after reaching the highest peak, even if the exposure to the news remained high. This is particularly evident in Estonia, Romania, Belgium, and the Czech Republic. Also, Latvia, Lithuania, and Poland have similar temporal profiles, presenting multiple peaks in both Wikipedia pageviews and the volume of news during the reporting time of ASF outbreaks. The communication of official reports of the outbreaks is not always reflected in the news volume, failing to trigger an information-seeking behavior towards the subject. This is particularly evident in Italy where, despite multiple ASF cases being reported before October 2018, Wikipedia searches only started after the first peak of news volume. Moreover, given the endemic state of ASF in the Sardinia region of Italy, the disease might be already known in the country, failing to trigger the information-seeking behavior in the timespan considered. These trends suggest a necessary threshold of information exposure to trigger the information-seeking behavior which is not necessarily dependent on the surveillance reports of the outbreaks.

**Table 1:** Pearson and Spearman correlation between Wikipedia pageviews and news volume.

Wikipedia—News by source country	Pearson	Spearman
ITALY	0,55	0,6
SOUTH KOREA	0,75	0,7
LATVIA	0,42	0,22
LITHUANIA	0,53	0,23
POLAND	0,53	0,72
ROMANIA	0,64	0,71
UKRAINE	0,33	0,46
CZECH REPUBLIC	0,57	0,79
ESTONIA	0,65	0,23
BELGIUM	0,86	0,71
CHINA	0,74	0,73
INDIA	0,4	0,72

In Table 2 we show the results of the two regressions models and in Table 3 we show the coefficient estimates for the model with the memory parameter.

**Table 2:** Adjusted R^2^ for the two linear regression models applied to predict Wikipedia visits.

Countries	News	News+Memory
ITALY	0.57	0.72
SOUTH KOREA	0.57	0.73
LATVIA	0.44	0.59
LITHUANIA	0.46	0.53
POLAND	0.36	0.37
ROMANIA	0.47	0.59
UKRAINE	0.51	0.61
CZECH REPUBLIC	0.43	0.48
ESTONIA	0.56	0.61
BELGIUM	0.78	0.79
CHINA	0.65	0.65
INDIA	0.28	0.64

**Table 3:** Coefficient estimates for the model with memory.

Countries	News	News+Memory
ITALY	0.20 [0.03, 0.37]	0.14 [0.11, 0.17]
SOUTH KOREA	0.41 [0.16, 0.65]	0.43 [0.19, 0.68]
LATVIA	0.17 [0.00, 0.34]	0.08 [0.06, 0.11]
LITHUANIA	0.41 [0.04, 0.78]	0.09 [0.04, 0.13]
POLAND	0.29 [-0.05, 0.62]	-0.05 [-0.21, 0.11]
ROMANIA	0.19 [-0.13, 0.51]	0.25 [0.16, 0.35]
UKRAINE	0.30 [0.14, 0.46]	0.34 [0.25, 0.43]
CZECH REPUBLIC	0.25 [0.07, 0.43]	0.16 [0.04, 0.27]
ESTONIA	0.25 [0.03, 0.48]	0.16 [0.10, 0.23]
BELGIUM	0.61 [0.36, 0.85]	0.09 [-0.03, 0.20]
CHINA	0.49 [0.17, 0.80]	-0.07 [-0.22, 0.09]
INDIA	-0.07 [-0.23, 0.08]	0.36 [0.20, 0.53]

The coefficients are all significant with *p<0*.*001*, except for Poland (*p = 0*.*009*), Romania (*p = 0*.*013*), Belgium (*p = 0*.*003*), and China (*p = 0*.*02*).

We compared the two models by using the adjusted coefficient of determination (R^2^) (Miles, 2005). We found that memory effects improve the model performance. Additionally, we compared the two models using the F-test for nested models [24], obtaining *p<0*.*001* in most of the cases except for Poland, Belgium, and China, for which *p<0*.*02*. Hence, strong statistical evidence suggested that adding the memory term improves the performance.

**Fig 2:**
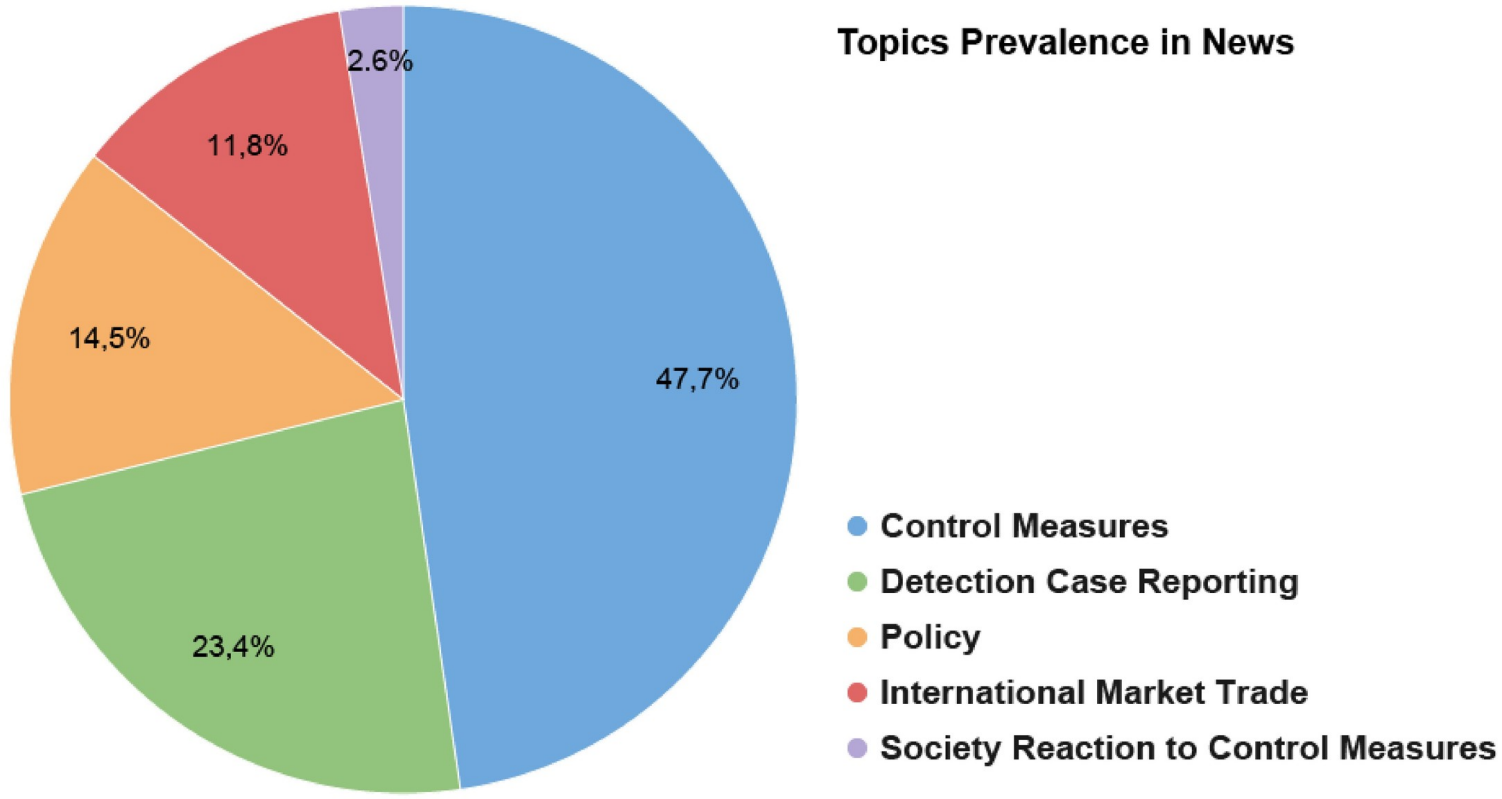
Percentage of documents per topic defined.

The digital news analyses led originally to twenty-five topics which were lately grouped into 5 main broad topics. Fig 2 shows the main topics in the Estonian news datasets. The most relevant one/ones refer/refers to control measures, intended to prevent both the spreading of ASF and the presence of infected products on the market. This result confirms the most prevalent topics exposure for Estonia to be about control measures during the entire period of the study. Although we cannot classify which proportion of the reached population are farmers or authorities, we can assume, given the specificity of the ASF topic, that they are a part of it. The exposure to control measures information is confirmed also by the analysis of the survey to the veterinary authorities.

### Questionnaires in Estonia to pig farmers and veterinary authorities

All pig farmers that participated in the focus groups discussions were invited to fill in the questionnaire. All pig farmers agreed to fill in the questionnaire (response rate of 100%). A total of 22 pig farmers answered the questionnaire. Demographic information regarding the farm’s counties can be found in (S4.1 Table in S1 File). Out of the 22 respondents, 4 (18.2%) had a multiplier production type, 7 (31.8%) kept fattening pigs and 11 (50%) managed a farrow-to-finish farm type. A total of 18 (81.8%) farmers declared that ASF has never been detected in their farms against 4 (18.2%) that mentioned that it was detected. Also, 17 (77.3%) farmers felt confident in recognizing the clinical signs of ASF in pigs and 5 (22.7%) did not. All farmers mentioned that they have invested resources in improving the biosecurity of their farms because of the ASF epidemic in Estonia. The five main biosecurity aspects in which farmers have invested resources included training staff on disease management (20/22, 90.9%), fencing (20/22, 90.9%), cleaning and disinfection (18/22, 81.8%), management of dead animals (17/22, 77.3%) and transport of animals (17/22, 77.3%). S4.2 Table in S1 File contains all the biosecurity measures in which Estonian farmers invested resources due to ASF. All farmers were aware that ASF is currently spreading in other countries. The five most selected sources to access information on ASF were social media (14/22, 63.6%), other internet sources (non-social media) (13/22, 59.1%), colleagues/friends (13/22, 59.1%), agricultural magazines (12/22, 54.5%), general papers/magazines (12/22, 54.5%). Farmers were also asked about the use of social media as a tool to share information on topics related to ASF. From a predefined list of possible answers provided, half of the farmers (11/22, 50%) reported that social media is useful to share information on ASF related topics, eight farmers (36.4%) mentioned that they prefer using other communication channels, and three (13.6%) that they do not normally use social media.

Regarding the farmers’ perception of the implementation of the ASF strategy, most farmers (21/22, 95.5%) felt well-informed about the measures recommended by the veterinary authorities to prevent/control ASF outbreaks in pigs. Similarly, most farmers (17/22, 77.3%) thought that there is cooperation between the farmers’ community and the veterinary authorities. Also, 15 out of the 22 farmers (68.2%) thought that farmers are sufficiently consulted by the veterinary authorities in terms of ASF control and 17 (77.3%) agreed with most of the measures to prevent/control outbreaks of ASF in pigs. Out of the 22 farmers, 17 (77.3%) agreed with most of the measures to prevent/control outbreaks of ASF in pigs and 5 (22.7%) did not. However, 16 out of 22 farmers (72.7%) did not feel satisfied with the economic support from the government to control ASF.

We assessed the level of satisfaction of the farmers (very dissatisfied, slightly dissatisfied, slightly satisfied, and very satisfied) regarding ASF communication practices of veterinary authorities considering two aspects: the communication promptly to farmers on ASF-related topics during an outbreak, and the content of the communication to farmers on ASF-related topics during an outbreak. We avoided using the “neutral” category in the satisfaction scale to force respondents to provide a rather positive (i.e., “slightly satisfied”—low positive satisfaction) or negative answer (i.e., “slightly dissatisfied”—low negative satisfaction). About fifty-four percent (54.5%, 12/22) of respondents were slightly satisfied, 27.3% (6/22) slightly dissatisfied, 13.6% (3/22) very satisfied, and 1 respondent was very dissatisfied with the timely manner of the communication to them on ASF-related topics during an outbreak. Half of the respondents (11/22, 50%) were slightly satisfied and 31.8% (7/22) were slightly dissatisfied and 18.2% (4/22) were very satisfied with the content of the communication on ASF-related topics directed to farmers during an outbreak. Also, 45.5% (10/22) of respondents felt slightly dissatisfied, 36.4% (8/22) were slightly satisfied, 9.1% (2/22) were very dissatisfied, and one respondent was very satisfied with the farmers’ involvement of the farmers’ community in the national ASF control strategy. Regarding the implementation of actions by the veterinary and other veterinary authorities promptly during an ASF outbreak, 45.5% (10/22) felt slightly satisfied, 31.8% (7/22) slightly dissatisfied, 13.6% (3/22) very satisfied, and 9.1% (2/22) very satisfied.

Eleven interviews were conducted with representatives of the Estonian veterinary authorities, three working at the central level and eight respondents working at the district level for the Estonian competent animal health authorities. Interviewees were asked which main institutions and private organizations were involved in the risk management strategy of ASF in domestic pigs and wild boars in Estonia. S4.3 Table in S1 File, shows the name and the number of nominations of public institutions and private organizations mentioned by respondents. Interviewees were asked about which target groups, communication channels, and materials are used as part of the ASF communication strategy. Their answers can be found in the S4.4 Table in S1 File. The coordination between public institutions and private organizations was described as good for most respondents (8/11, 72.7%), adding that it has improved from the beginning of the epidemic until the moment that the interview was taken. Most of the barriers encountered in the coordination mentioned by respondents were related to communication aspects (47.4%), lack of direct funding to support coordination (15.8%), poor commitment (15.8%), lack of clear chain of command (10.5%) and lack of transparency (10.5%).

The coordination between the district and central veterinary authorities was positively assessed by respondents (9/11, 81.8%). Only one respondent assessed this coordination as dissatisfactory, and the other respondent did not answer this question.

## Discussion

SARS-CoV-2 has shown us that public health emergencies are to be managed at the general population level as well as at the local level and these two approaches must be synergic and be driven by a common goal. Some public health emergencies may have devastating impacts on the economy and all efforts should be made to limit the spread of emerging or re-emerging pathogens, including the support that can be offered by the general population. ASF is a perfect example to investigate as disease control measures rely on combating the risk of introduction and spread by applying measures at a farm level, at the national level, and at the level of the general public (e.g. citizens being recommended on appropriate individual behaviors, such as proper disposal of food in recreational areas and accessible trash disposal units, and help implement passive surveillance such as reporting of wild-boar carcasses from hunters and tourists to authorities). The reason for this is that urban sprawl has become common in certain parts of Europe due to the increase in wild-boar demographics and the availability of anthropogenic food sources [39, 40] such as trash and leftover food from pets. In addition, the scavenging behavior of wild boars toward their dead fellows is believed to be a means by which ASF can be amplified, and thus immediate removal of carcasses is believed to be an important means of controlling the spread of ASF [41]. Engagement of the general public is essential to mitigate the risk of infection and that preventative behavior such as prohibiting swill feeding and pork product release into the environment and early warning systems such as immediate reporting to authorities of dead animals can be essential when the disease is actively circulating. In this study, we attempt to tackle these diverse scales of disease awareness and preparedness by combining non-traditional data sources (i.e. digital traces generated by the activity of users on digital platforms) and more traditional data collection approaches to assess the information-seeking behavior as well as the awareness and the preparedness concerning ASF at two different scales (general population and target groups) intending to evaluate the applicability of an integrated approach to monitor public awareness and preparedness of health authorities and field workers based on a combination of non-traditional data sources and survey-based methodologies.

In the case of ASF, specific sub-groups of the population that are at the forefront of an epidemic preparedness, such as pig farmers, differ from the general population in the sense that they are considerably more aware and interested in a topic that affects them directly, and this is reflected in the results of the questionnaire. Not the same can be said for the general population, given that digital information-seeking behavior peaked only after generalist news coverage. For these reasons, both approaches (digital and field-oriented) are valuable and needed from a public health perspective to understand how interest, risk perception, and awareness in the general population and specific interest groups evolve during an epidemic and how they may affect public opinion as well as the preparedness.

To achieve this, first, we exploited the pervasiveness of digital data to assess the awareness among the general population as measured through Wikipedia page views in reaction to the exposure to news about ASF outbreaks on an international scale. Then, we examined the preparedness, awareness, and information-seeking in localized areas through questionnaires to farmers and health authorities, with a focus on the Estonian context. Estonia had experienced an extensive epidemic of ASF among wild boar as well as in domestic pigs from 2014 to 2017 resulting in significant economic consequences to the pig farming industry [42]. The wild boar population decreased several folds due to the disease spread and intensified hunting [43]. At the same time, there is a strong digital infrastructure in Estonia and internet-based services are widely used for governance and communication [44] making Estonia suitable for this study.

Expectedly our results show that, at an international scale, public interest peaks rapidly during an initial attention window which occurs after exposure to news coverage of a specific outbreak. In particular, the public activity profile as measured through the access to the Wikipedia pages shows non-linear dependencies and memory effects in the relation between information seeking, media pressure, and disease dynamics. These peaks of attention rapidly decline as media outlets drop the topic. This pattern of information-seeking behavior can be an important clue that needs further investigation. We cannot know the multiple reasons that lead the general public to look for information on ASF in Wikipedia. However, this Wikipedia search behavior could be the basis to establish a means of providing relevant guidance to information seeking users on how they can contribute to reducing the risk of human-mediated spread of infection and on how they may contribute both to passive surveillance efforts and to reducing the risk of spread from carcasses. It brings an opportunity to inform and build a dialogue between citizens and policymakers involved in disease control efforts.

This suggests that if governmental and health authorities’ outreach strategies become dynamic and customized to the evolving epidemiological situation, a bottom-up response coming from the general public could become a tool to support control and mitigation efforts. This opportunity may have important public health implications. Consistently with previous literature work for different human diseases and zoonotic outbreaks [22, 24], we found that Wikipedia page activity is highest in correspondence of media coverage of official communications by public health authorities. This is particularly relevant because official communications amplified by media coverage could effectively be leveraged to capture the public attention and stimulate information-seeking even in a longer-term after the outbreak.

This is confirmed by the topic discovery activity, news articles covered a broad range of topics: control measures, detection, case reporting, and policy. In particular, these have been the main topics on the news coverage in Estonia in addition to “society reaction to control measures”. This points to the fact that, in the specific case of Estonia, the media have been actively engaging the general population specifically affected by the problem of ASF.

The focus on Estonia has been further explored through the field data collection (surveys proposed to farmers and veterinary authorities) which allowed us to assess communication practices in a country that was heavily affected by the disease [13]. The focus on communication practices and access to information from farmers is innovative as these aspects are not extensively and regularly captured in field surveys aiming to assess the risk management strategies. Most farmers felt well-informed about the measures recommended by the veterinary authorities to prevent/control ASF outbreaks in pigs. Similarly, most farmers thought that there is cooperation between the farmers’ community and the veterinary authorities. This is a finding of relevance because usually, farmers tend to complain against veterinary authorities because of culling practices and poor compensation policies [11, 13]. In our sample, despite having 16 out of 22 farmers not satisfied with the compensation, the majority was still positive about the risk management strategies implemented. Moreover, farmers reported being satisfied with the timeliness of communication on ASF by the animal health authority during an outbreak. This points to a positively evolving relationship as, in general, there is the perception among farmers that the authority could have been more efficient when it comes down to infectious disease management [45].

Importantly, the coordination between public institutions and private organizations was described as good for most respondents, adding that it has improved from the beginning of the epidemic until the moment that the interview was taken. The coordination between all these actors (public and private) is known to be crucial for disease control. Hunters, farmers, and private veterinarians’ associations are key players in ASF risk management strategies and, therefore, coordination among these actors is extremely relevant. Our study shows that the timeliness of transmitting official communication between districts and the central level during an ASF outbreak was referred to as “satisfactory” by about half of the respondents. This is relevant since the epidemiological situation is evolving and may be subject to change and thus require revised control strategies. Finally, almost all the respondents mentioned that there is cooperation between farmers/hunters and the veterinary authorities to control ASF and this certainly has facilitated keeping ASF mostly under control in Estonia.

On the other hand, among the compliance issues mentioned by the animal health authority respondents (lack of financial support and knowledge, cooperation and commitment of farmers and hunters, lack of detailed ASF legislation, lack of regular training and equipment), lack of support from the press was also mentioned. This is in line with the information-seeking behavior that we observed by looking at the amount of news devoted to outbreaks and the expected declining attention level from the general public once the news coverage is over.

Finally, the local health authorities identified difficulties in addressing specific preventative aspects which are related to ASF such as reaching moving target groups (e.g., travelers, truck drivers) and keeping them engaged. It is vital to engage these categories of people given the transboundary nature of ASF, recently highlighted by the outbreak in the Dominican Republic spreading within a couple of months to Haiti [46] and thus becoming a tangible threat to the pork industry of the Americas [47]. In previous outbreaks travelers/truck drivers, foreign workers were suspected of being involuntarily involved in the introduction of the disease in Belgium [48], and for this reason, additional control measures such as garbage disposal from planes coming from selected infected areas are being implemented in the US. To meet the needs of increased prevention strategies, a targeted engagement campaign, coordinated by the authority with the support of private organizations, including the provision of web-based information could be developed to reduce the risk of transboundary spread. As an example, basic information is provided on relevant Wikipedia pages or other non-scientific web sources (i.e., an information website for tourists such as TripAdvisor) such as recommendations to avoid traveling abroad with any pork products and immediately inform authorities if noticing wild boar carcasses would support such efforts. The results of this study highlight the potential strength of combined conventional and digital multi-stakeholder engagement efforts to contribute to reducing the spread and impact of potentially devastating epidemics of transboundary animal diseases such as ASF.

### Limitations

During the study, we faced several limitations, especially with field activities that were complicated by the concurrent SARS-CoV-2 pandemic on multiple fronts. These included the small sample size of both Estonian farmers and veterinary authorities. Only 22 out of 30 farmers (the initial target) have responded to the questionnaire. The disruption of the data collection process was caused to SARS-CoV-2 restrictions on travel hampering the implementation of the survey.

A way to overcome the logistic limitations imposed by field surveys, the implementation of digital surveys for both sectorial workers and the general population could bring considerable benefits. This would extend the reachable countries and provide guidelines for the implementation of optimal country-specific strategies to improve awareness for both sectorial workers and the general population.

Also, the fact that Estonian pig farmers filled in the questionnaire after focus group discussions on ASF control measures might have brought anchoring bias, affecting their answers to the questionnaire.

## Future work

Possible future directions for this work would focus on creating a user-friendly digital version of the surveys that could be regularly used by authorities aimed at reaching out to a larger number of farmers and health authorities in a multi-country fashion. Along these lines—and concerning the evolving epidemiological situation—developing a format that could allow “living questionnaires” would represent an innovative tool to manage established epidemic situations. This could be relevant for ASF given that ASF notifications do not appear to be declining in many countries but could also apply to many other hazards with relevant public health impacts. This could also represent a means of engaging the population categories which can be affected by the problem of ASF both through content delivery and to encourage active participation in the collection of information.

## Supporting information

S1 Fig (TIF)

S1 Table Total pageviews and news for the selected countries. (XLSX)

S1 File Linear regression-diagnostics. (DOCX)

S2 File Questionnaire to Estonian farmers. Selected questions analyzed in this study are marked with a star*.
(DOCX)

S3 File Questionnaire to the Estonian veterinary authorities. Selected questions analyzed in this study are marked with a star*.
(DOCX)

S1 Text GDELT query for data collection. (DOCX)

S1 Data Timeseries data from news, Wikipedia, and outbreaks for swine and wild boar. (ZIP)
