# Supplementary figures and images for: Integrating digital and field surveillance as complementary efforts to manage epidemic diseases of livestock: African swine fever as a case study
Source: PLoS One. 2021 Dec 31;16(12):e0252972. doi: 10.1371/journal.pone.0252972 (PMC8719698; doi:10.1371/journal.pone.0252972)

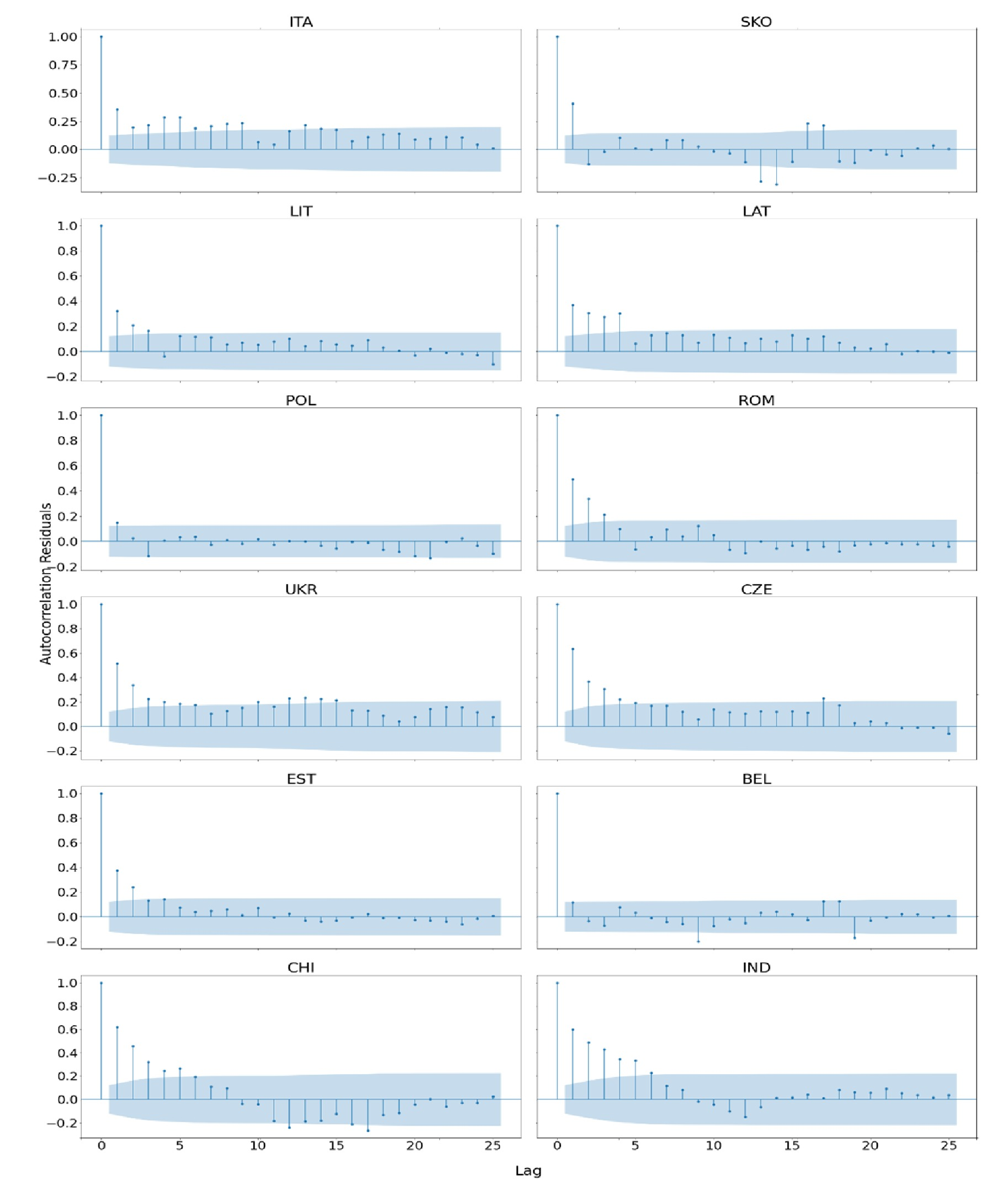

Supplement: S1 Fig — (TIF) [file pone.0252972.s001.tif]
