# Supplementary material for: Integrating digital and field surveillance as complementary efforts to manage epidemic diseases of livestock: African swine fever as a case study
Source: PLoS One. 2021 Dec 31;16(12):e0252972. doi: 10.1371/journal.pone.0252972 (PMC8719698; doi:10.1371/journal.pone.0252972)
Supplement: S1 File — (DOCX) [file pone.0252972.s003.docx]

## S2. Linear Regression – Diagnostics

In the main text, we use a Liner Model to nowcast the amount of Wikipedia page views media coverage as independent variables. Here, we test model assumptions of homoscedasticity and no-autocorrelation of residuals. Homoscedasticity of residuals is assessed with the Breusch-Pagan Lagrange Multiplier test (Breusch & Pagan, 1979) on a fitted Ordinary Least Square model (OLS) using python statsmodels library (https://www.statsmodels.org/). Assuming a significance level $\alpha=0.05$, we obtain heteroscedasticity of residuals for Model II and Model III (S2 Table).

S2 Table. P-values for the Breusch-Pagan Lagrange Multiplier test on heteroscedasticity. The null hypothesis is that residuals are homoscedastic, hence a p-value < 0.05 indicates heteroscedasticity.

| Countries | Model I | Model II |
| --- | --- | --- |
| Italy | 0.0 | 5.3E-07 |
| South Korea | 0.0 | 6.7E-35 |
| Lithuania | 0.0 | 1.2E-09 |
| Latvia | 0.0 | 1.9E-21 |
| Poland | 0.0 | 3.9E-17 |
| Romania | 0.0 | 1.1E-21 |
| Ukraine | 0.0 | 4.4E-09 |
| Czech Republic | 0.0 | 9.8E-11 |
| Estonia | 0.0 | 2.7E-13 |
| Belgium | 0.0 | 1.0E-17 |
| China | 0.0 | 4.1E-21 |
| India | 0.0 | 1.2E-10 |

We check for the autocorrelation of residuals in S2 Figure, where we plot the partial auto-correlation function for Model II (analogous results are obtained for Model I). We notice a significant correlation at the first four lags in all cases, and in some cases also at higher orders.

S2 Figure Auto-correlation function for Model II. In the first four lags, we notice a significant correlation for all the countries.
