# Supplementary material for: Integrating digital and field surveillance as complementary efforts to manage epidemic diseases of livestock: African swine fever as a case study
Source: PLoS One. 2021 Dec 31;16(12):e0252972. doi: 10.1371/journal.pone.0252972 (PMC8719698; doi:10.1371/journal.pone.0252972)
Supplement: S2 File — Selected questions analyzed in this study are marked with a star*. (DOCX) [file pone.0252972.s004.docx]

## S3 Questionnaire to Estonian farmers.

**SECTION 1: Demographic information**

1. *Give the name of the County and City/Village where your farm is located.

County_________

City/Village _________

**SECTION 2: Farm characteristics**

1. *What is your type of production? Select one answer.

| - (1)Multiplier^^[[1]](#footnote-1)^^ | - (3)Farrow-to-finish^^[[2]](#footnote-2)^^ | |
| --- | --- | --- |
| - (2)Fattening^^[[3]](#footnote-3)^^ |  | |
|  |  |  |

**SECTION 3: Experience related to African Swine Fever (ASF)**

1. *Has ASF ever been detected in your farm? Select one answer.

| - No - Yes | - I do not know |
| --- | --- |

1. *Do you feel confident that you can recognise the clinical signs of ASF in pigs? Select one answer.

| - No - Yes |  |
| --- | --- |

1. *Have you invested resources in improving the biosecurity of your farm (equipment, practices, vehicles, facilities etc.) as a result of outbreaks of ASF? Select one answer.

| - No (Go to question 8) - Yes (Go to question 7) |  |
| --- | --- |

1. *In what aspects of biosecurity did you invest resources in your farm due to ASF?

| - (1)Feed, water | - (7)Management of dead animals |
| --- | --- |
| - (2)Equipment supply | - (8)Training staff on disease management |
| - (3)Transport of animals | - (9)Cleaning and disinfection |
| - (4)Removal of manure | - (10)Constructional changes |
| - (5)Fencing | - (11)Other___________________ |
| - (6)Sourcing of animals |  |

8. *Do you think that ASF is currently spreading in other countries? Select one option.

| - No (Go to question 12) - Yes (Go to question 11) |
| --- |

9. *Where did you learn this? Check all that apply.

| - (1)Social media^^[[4]](#footnote-4)^^ | - (5)General papers/magazines |
| --- | --- |
| - (2)Other internet resources (non-social media) | - (6)Agricultural magazine |
| - (3)Colleagues/friends | - (7)Other______________ |
| - (4)Local news media (TV –radio) |  |

1. *What do you think about the use of social media to share information on topics related to ASF? Select all that apply.

- (1)Social media are useful to share information on topics related to ASF.
- (2)I prefer sharing information on ASF using other communication channels.
- (3)I do not normally use social media so I do not have an opinion about this question.

**SECTION 4: Perception of the implementation of the strategy on African Swine Fever (ASF)**

1. Select one answer for each of the following questions:

| - *(1)Do you think that there is cooperation between the farmers’ community and the authorities to prevent/control ASF? | - Yes | - No |
| --- | --- | --- |
| If No, please, specify briefly why: | | |
| - *(2)Do you think that farmers are sufficiently consulted by the authorities when it comes to prevent/control ASF? | - Yes | - No |
| If No, please, specify briefly why: | | |
| - *(3)Are you satisfied with the economic support from the government to control ASF? | - Yes | - No |
| If No, please, specify briefly why: | | |
| - *(4)Do you feel you are well-informed about the measures recommended by the authorities to prevent/control outbreaks on ASF in pigs? | - Yes | - No |
| If No, please, specify briefly why: | | |
| - *(5)Would you say that you agree with the majority of the measures to prevent/control outbreaks on ASF in pigs? | - Yes | - No |
| If No, please, specify briefly why: | | |

1. What is your level of satisfaction with the veterinary and other authorities in relation to ASF? Tick one option for each of the following aspects.

| Aspects: | Very satisfied | Slightly Satisfied | Slightly Dissatisfied | Very dissatisfied |
| --- | --- | --- | --- | --- |
| *(1)Involvement of the farmers’ community in the national ASF control strategy |  |  |  |  |
| *(2)Communication in a timely manner to farmers on ASF-related topics during an outbreak |  |  |  |  |
| *(3)Content of the communication to farmers on ASF-related topics during an outbreak |  |  |  |  |
| *(4)Implementing actions in a timely manner during an ASF outbreak |  |  |  |  |

##

##

1. Multiplier: Pig herd formed by breeding animals and piglets (up to weaned pigs) [↑](#footnote-ref-1)
2. Farrow-to-finish: Pig herd that comprises all the categories of pigs. [↑](#footnote-ref-2)
3. Fattening: Pig herd consisted of fattening pigs. [↑](#footnote-ref-3)
4. Social media: websites and applications to share information and be part of social networking (e.g.Facebook, Instagram, LinkedIn etc...) [↑](#footnote-ref-4)
