# Supplementary material for: Integrating digital and field surveillance as complementary efforts to manage epidemic diseases of livestock: African swine fever as a case study
Source: PLoS One. 2021 Dec 31;16(12):e0252972. doi: 10.1371/journal.pone.0252972 (PMC8719698; doi:10.1371/journal.pone.0252972)
Supplement: S3 File — Selected questions analyzed in this study are marked with a star*. (DOCX) [file pone.0252972.s005.docx]

## S4 Questionnaire to the Estonian veterinary authorities.

**SECTION 1: General**

1. *First, could you please tell me about your **job position** and **responsibilities** in your organization when it comes to ASF?

*Please, tell me more…*

**SECTION 2** **: Coordination practices related to the national risk management of African Swine Fever (ASF)**

**2-** *Could you please tell me which are the **main institutions and organizations (public and private)** actively involved in the risk management strategy of ASF (in domestic pigs (DP) and wild boars (WB) in Estonia?

3- *How would you **describe the coordination** between these institutions? From your perspective, which are the **main barriers** encountered in the coordination?

*Why do you say that…?*

*Potential barriers to mention:*

- 1. *Communication*
  2. *Transparency*
  3. *Lack of clear chain of command*
  4. *Poor commitment from people at different level*
  5. *Language (use of terminology)*
  6. *Lack of direct funding to support coordination*
  7. *Others. Please add:…………………………………………*

*4-* *How would you assess that is the **coordination** between the regional/**district** and national/**central veterinary authorities**?

- 1. *Very satisfactory*
  2. *Slightly satisfactory*
  3. *Satisfactory*
  4. ***Slightly dissatisfactory***
  5. ***Dissatisfactory***
  6. ***Very dissatisfactory***

*In case of A/B/C,*

- *What specifically do you find satisfactory?*

*In case of D/E/F,*

- *What specifically do you find dissatisfactory?*

**SECTION 3** **: National communication strategy on African Swine Fever (ASF)**

I would like to move into the national **communication strategy** on ASF, based on your knowledge and experience.

5. *Which are the **main** target groups of the communication strategy of ASF?

*Potential target groups:*

| *Smallholder pig keepers* |  | *Hunters* |  |
| --- | --- | --- | --- |
| *Commercial farmers* |  | *Forest workers* |  |
| *Temporal farm workers* |  | *Veterinarians* |  |
| *Pig-breeders* |  | *Transport authorities and check point staff* |  |
| *Livestock transporters* |  | *General public (esp. travellers)* |  |
| *Slaughterhouse workers* |  |  |  |

6. *Which are the **main** communication channels used for those target groups?

7.- *What would you describe as the **shortcomings** of the current communication strategy?

*Why do you say that…?*

*Shortcoming: deficiency, weak points*

S4.1 Table: Number of farmers interviewed by county (Estonian region).

| County | Number | Frequency (%) |
| --- | --- | --- |
| Lääne-Viru | 5 | 22.7 |
| Tartu | 3 | 13.6 |
| Jõgeva | 4 | 18.2 |
| Põlva | 1 | 4.5 |
| Harju | 2 | 9.1 |
| Järva | 1 | 4.5 |
| Saare | 5 | 22.7 |
| Ida-Viru | 1 | 4.5 |
| Total | 22 | 100 |

S4.2 Table: Biosecurity aspects that Estonian farmers mentioned to have invested resources due to ASF.

| Question | Category | Number of responses | Percentage (%) |
| --- | --- | --- | --- |
| In what aspects of biosecurity did you invest resources in your farm due to ASF? | Cleaning and disinfection | 18 | 81.8 |
|  | Constructional changes | 13 | 59.1 |
|  | Equipment supply | 8 | 36.4 |
|  | Feed, water | 9 | 40.9 |
|  | Fencing | 20 | 90.9 |
|  | Management of dead animals | 17 | 77.3 |
|  | Other | 1 | 4.5 |
|  | Removal of manure | 7 | 31.8 |
|  | Sourcing of animals | 6 | 27.3 |
|  | Training staff on disease management | 20 | 90.9 |
|  | Transport of animals | 17 | 77.3 |

S4.3 Table: Public institutions and private organizations mentioned by the Estonian veterinary authorities as part of the risk management strategy.

| Public Institutions | Nominations |
| --- | --- |
| Veterinary and Food Board | 9 |
| Veterinary and Food laboratory | 7 |
| Environmental Board | 7 |
| Ministry of Rural Affairs | 5 |
| University of Life Sciences | 5 |
| Estonian environmental agency | 3 |
| Ministry of environment | 2 |
| Rendering plant | 2 |
| Tax and custom board | 1 |
| Environmental inspectorate | 1 |
| Police | 1 |
| Private organizations | **Nominations** |
| Estonian hunters’ association | 9 |
| Pig farmers’ association | 4 |
| Private veterinarians | 2 |
| Pig breeder society | 1 |

S 4.4 Table. Target groups, information material and communication channels mentioned by the Estonian veterinary authorities

| **Target group** | Communication channels | | |  |
| --- | --- | --- | --- | --- |
|  | **Electronic channels** | **Written methods** | **Face-to-face communication** | **Broadcast media communication** |
| Smallholder pig keepers | Website of the ministry of rural affairs, emails, posters, leaflets | Posters, leaflets, newspaper articles | Meetings |  |
| Commercial farmers | Website of the ministry of rural affairs, emails | Posters, leaflets, newspaper articles | Meetings |  |
| Temporal farm workers | Website of the ministry of rural affairs |  |  |  |
| Pig-breeders | Website of the ministry of rural affairs, posters, leaflets | Posters, leaflets, newspaper articles |  |  |
| Livestock transporters | Website of the ministry of rural affaires |  |  |  |
| Slaughterhouse workers | Website of the ministry of rural affairs |  | Oral presentations of official veterinarians to workers |  |
| Hunters | Website of the ministry of rural affairs, posters, leaflets, emails with information updated for the district | Magazine from the hunter’s association, posters, leaflets, newspaper articles | Meetings with the veterinary and food board |  |
| Forest workers | Website of the ministry of rural affairs |  |  |  |
| Veterinarians | Website of the ministry of rural affairs |  |  |  |
| Transport authorities and check point staff | Website of the ministry of rural affairs, posters | Posters |  |  |
| General public | Website of the ministry of rural affairs, posters | Posters, newspapers articles |  | News on the TV/radio, Facebook page of the veterinary and food board |
| Military forces/government rescue service | Website of the ministry of rural affairs, NETO guidelines about biosecurity rules in the environment |  | Military events | Public news about biosecurity to soldiers |
| Feed producers | Website of the ministry of rural affairs, emails |  |  |  |
| Official vets in meat plants |  |  | Training courses |  |
