# Supplementary material for: Integrating digital and field surveillance as complementary efforts to manage epidemic diseases of livestock: African swine fever as a case study
Source: PLoS One. 2021 Dec 31;16(12):e0252972. doi: 10.1371/journal.pone.0252972 (PMC8719698; doi:10.1371/journal.pone.0252972)
Supplement: S1 Text — (DOCX) [file pone.0252972.s006.docx]

S5 GDELT Query for ASF news

The data were collected using from the GDELT database using Google Bi Query ( https://cloud.google.com/bigquery) through the following query.

SELECT DocumentIdentifier, DATE, V2THEMES, V2Organizations, V2Persons, V2Locations, TranslationInfo
FROM `gdelt-bq.gdeltv2.gkg_partitioned`
WHERE _PARTITIONTIME >= "2015-01-01 00:00:00"
AND ((DocumentIdentifier LIKE '%swine%fever%') or (DocumentIdentifier LIKE '%influenza%suina%')
 or (V2THEMES LIKE '%TAX_DISEASE_AFRICAN_SWINE_FEVER%'))
